# *Caulobacter* Strains Code for Novel Restriction Endonucleases That Protect Them from Bacteriophage Infections

**DOI:** 10.3390/v17030311

**Published:** 2025-02-25

**Authors:** Ian Sisto, Bert Ely

**Affiliations:** Department of Biological Sciences, University of South Carolina, Columbia, SC 29208, USA; ian.sisto@gmail.com

**Keywords:** *Caulobacter*, restriction enzymes, phage plating efficiency, bacteriophage, DNA methylation

## Abstract

Bacteriophages grown on *Caulobacter vibrioides* strain CB15 have reduced plating efficiency on other *Caulobacter* strains. To determine the cause of this reduced plating efficiency, we performed a series of experiments that demonstrated that the reduced plating efficiency is due to a novel set of restriction and modification (RM) enzymes that are present in most of the *Caulobacter* strains that we tested. We then demonstrated that one of these RM systems recognizes the nucleotide sequence 5′-ATNNAT-3′. A careful inspection of the genome nucleotide sequences of each of the strains revealed that the genes coding for these RM enzymes have not been annotated or identified, suggesting that the proteins may differ from the common types of bacterial restriction and modification enzymes. In addition, the host strain NA1000 contains a 26 kb mobile element that provides resistance to incoming phages.

## 1. Introduction

Bacteriophages, also known as phages, are diverse types of viruses that infect bacteria. The phages code for their own enzymes and proteins to seize cellular resources to generate more phages. In the lytic cycle, the phage genome is expressed in the host cytoplasm and leads to the assembly of new phage particles and the lysis of the host cell [1]. Alternatively, in addition to the lytic cycle, some phages can use the lysogenic cycle where they incorporate their genomes into the host genome and remain dormant while the host bacterium reproduces normally. Thus, every time the host genome is replicated, the integrated phage genome is replicated as well. Phages are important in controlling the population levels of bacteria. In addition, phages impact bacterial evolution since they can transfer DNA from one bacterium to another. However, bacteria have evolved several kinds of defense mechanisms against phages. For example, bacterial mutations can change binding sites for phages and prevent the phages from attaching [2]. Other internal defense mechanisms, such as restriction and modification systems and cell suicide, are complex and defeat the phage once its genome enters the cell.

Restriction and modification systems are innate bacterial defense systems that directly seek and destroy phage genomes that enter the cytoplasm. These defense systems utilize two bacterial enzymes: a restriction endonuclease and a methlytransferase. These enzymes serve different functions but work together to protect the bacteria and eliminate the phage threat [3]. Restriction endonucleases target specific short DNA sequences and make double-strand cuts in the DNA. To prevent this same short DNA sequence from being cut in the bacterium’s own genome, the methlytransferase adds a methyl group to the C5 carbon of nucleotides in the target sequences that prevent restriction enzyme cleavage. Although both enzymes target the same nucleotide sequence, the restriction endonucleases bind more efficiently, so incoming DNA is usually cleaved before it can be methylated. The combination of restriction endonuclease and methylase enzymes is effective as phage DNA is often unmethylated and vulnerable to the more efficient restriction endonuclease cuts. Bacterial restriction enzymes have been classified as type I or type II based on the nature of their recognition and cleavage sites. Because restriction enzymes are so effective and sequence-specific, they have been widely used in biotechnology for molecular cloning, gene editing, PCR, and other molecular techniques [3].

*Caulobacter vibrioides* is a bacterial species with a complex life cycle. These bacteria have a stalk/swarmer life cycle where mature stalked bacteria generate immature swarmer bacteria with flagella that are motile [4]. The swarmer bacteria subsequently lose their flagella and mature into stalked bacteria. A type II RM system designated CcrI has been characterized in the *Caulobacter vibrioides* strain CB13 [5]. The CcrI restriction endonuclease recognizes the sequence 5′-CTCGAG-3′ and cleaves both strands of the sequence between the C and T residue. The genome of a second strain of *C. vibrioides* designated CB2 does not have the genes that code for the CcrI RM, but it does code for a type I RM system. This type I restriction enzyme is similar to the *E. coli* type I EcoKI enzyme that recognizes the AAC[N_6_]GTGC or GCAC[N_6_]GTT nucleotide sequences and cuts at a distant site. The CB2 genome also codes for a putative type II restriction endonuclease designated NmeDIP. However, no methylase is paired with this type II restriction endonuclease gene. Two additional strains of *C. vibrioides* designated CB1 and CB15 have genes that code for the same type I restriction system as strain CB2, but the annotations of their genome sequences do not predict the presence of genes that code for any other restriction enzymes [6,7].

*Caulobacters* are susceptible to numerous phages [8]. One of the most common types of *Caulobacter* phages is a group of giant phages with elongated heads and flexible tails belonging to the recently designated subfamily *Dolichocephaloviridae* (https://ictv.global/taxonomy, accessed on 1 September 2024). In addition, phages have a limited host range, and we have shown that many of them can infect only a few strains of *Caulobacter.* For example, phage JessA is able to infect *C. vibrioides* and *C. segnis* strains, while the KSC phage infects only *C. vibrioides* strains [9]. Perhaps because its genome does not code for any type II restriction enzymes, CB15 serves as a good host strain for bacteriophages [8]. In contrast, *Dolichocephaloviridae* phage plating efficiency is 2% to 20% with strain CB2 compared to the plating efficiency with the original host strain CB15 [10], suggesting the presence of an RM system in CB2 cells. Based on this observation and the presence of genes that code for RM in the CB2 and CB13 genomes, we hypothesized that both CB2 and CB13 have functional and efficient RM systems that methylate genomic DNA and defend the cells from phage DNA invasion. However, any phages that had been grown on CB2 as a host should have been packaged with methylated DNA that would protect against the CB2, RM system, and therefore, the same number of plaques should be obtained using either CB2 or CB15 as host strains. To examine this phenomenon further, we analyzed the plating efficiency of the ERS, KSC, and Lullwater phages using NA1000, CB15, CB1, CB2, and CB13 as host strains.

## 2. Materials and Methods

The *C. vibrioides* wild-type strains NA1000, CB1, CB2, and CB13 were used as phage host strains along with a streptomycin resistant derivative of *C. vibrioides* CB15 designated SC1004 [11]. Cultures of the host strains were grown overnight at 35 °C in PYE [12]. To determine phage titer, 100 μL of appropriate dilutions of a phage lysate and 100 μL of an overnight culture of the host bacterium were mixed with 3.5 mL of SSM (PYE plus 0.5% agar). After overnight incubation at 35 °C, phage plaques were observed and counted. Phages lysates were prepared in the same manner except that we used a dilution of a phage lysate that would generate several thousand plaques. After overnight incubation, 6 mL of PYE was poured onto the surface of the plate, and the plate was refrigerated overnight. The next morning, the surface liquid was decanted into a capped tube and mixed with a small amount of chloroform to kill any residual bacteria.

### 2.1. Formation of Agarose Plugs Containing Intact Phage Genomes

A 500 mL aliquot of a concentrated phage lysate resuspended in Pett IV (10 mM Tris, pH7.6 plus 1 M NaCl) was mixed with 500 mL of molten 1% PFGE agarose and drawn into a 1 cc syringe [13]. When the mixture in the syringe solidified, we cut off the end of the syringe and pushed out the mixture while cutting it into small pieces using a sterile blade. The pieces were added to a 15 mL tube containing 2 mL of lysis buffer and 200 μL of Proteinase K and incubated at 50 °C overnight. After the incubation, the fluid was removed, and 2 mL of TE buffer (10 mM tris, 1 mM EDTA, pH 8.0) with 30 μL of PMSF was added to inactivate the Proteinase K. After mixing on a rotator at room temperature for 1 h, this step was repeated. Afterwards, the fluid was removed and replaced with 2 mL of TE buffer and mixed for 1 h and then repeated twice more. After the final wash, the plugs in TE buffer were stored in a refrigerator at 4 °C.

Plugs containing intact bacterial genomes were prepared by spinning 1 mL of an overnight culture for 2 min in a microcentrifuge. The resulting bacterial pellet was resuspended in 1 mL of Pett IV, spun and resuspended a second time, and then mixed with melted PFGE agarose and processed in the same manner as the phage plugs.

### 2.2. Restriction Enzyme Digests

Restriction endonuclease digests contained 60 μL of deionized water, 10 μL of NEB Cutsmart buffer, 3 μL of restriction enzyme, and one DNA-containing plug and were incubated at 37 °C for 4 h or overnight. The digested plugs were then subjected to 1% agarose gel electrophoresis and then stained with ethidium bromide. Undigested chromosomal DNA stayed in the plugs in the wells of the gel, while chromosomal DNA, which had been cleaved by the restriction enzyme, moved from the plug into the gel.

## 3. Results

Based on previous results [10], we hypothesized that phages grown using CB15 as a host should have lower plating efficiencies when strains CB2 or CB13 were used as alternate hosts. When the plating efficiencies were examined, phage ERS grown on CB15 had a seven-fold lower plating efficiency on CB2 and a 10^−4^ plating efficiency on CB13 (Table 1). In contrast, ERS grown on CB2 or CB13 had similar plating efficiencies on CB2, CB13, or CB15, suggesting that growth on either CB2 or CB13 protected the phage DNA from subsequent REase cleavage. Phage KSC grown on CB15 had even greater reductions in plating efficiency when plated on CB2 and CB13 (Table 1), supporting the idea that restriction enzymes reduced unprotected phage plating efficiency. In contrast, phage Lullwater grown on CB15 had only minor reductions in plating efficiency when plated on CB2 and CB13 (Table 1), suggesting that this phage might use a different method to avoid restriction enzyme cleavage of its genome.

**Figure 1:**
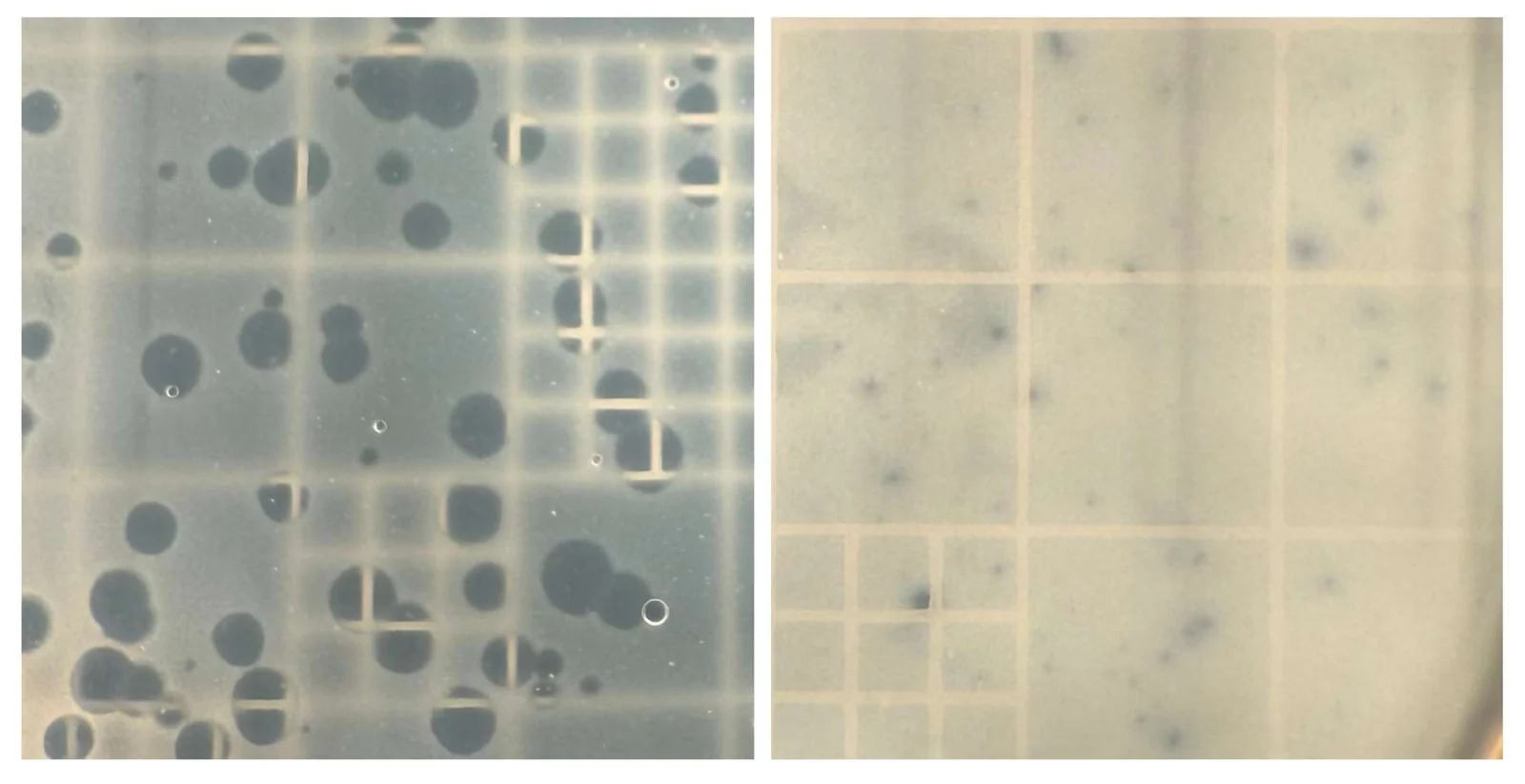
Phage ERS plaques with CB15 (**left**) or CB13 (**right**) as a host.

To determine if the observed reductions in plating efficiency were due to restriction enzymes, we grew lysates of each phage using either CB2 or CB13 as hosts. KSC and Lullwater phages grown on CB2 or CB13 had similar plating efficiencies on all three hosts, suggesting that the phage genomes had been modified (Table 1). ERS also had similar plating efficiencies on all three hosts except that the plating efficiency of ERS on CB13 was 34 times higher when ERS was grown on CB13. We also observed that phage plaques were two to ten times larger when any of the phages were plated on CB15 regardless of which strain was used as the previous host (Figure 1).

### Genomic Analysis

**Figure 2:**
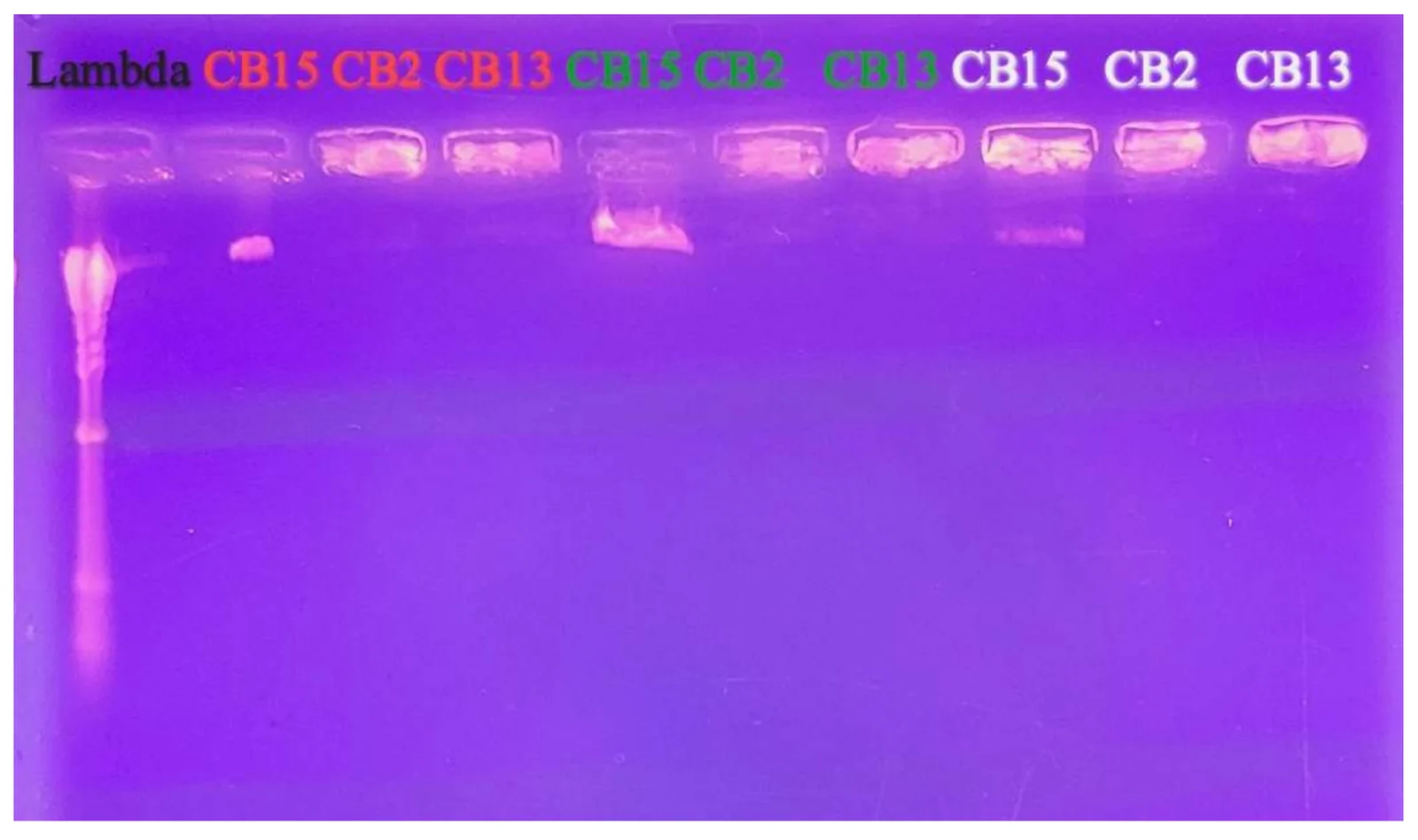
Gel electrophoresis performed on NsiI-digested CB15, CB2, and CB13 (red), BspDI-digested CB15, CB2, and CB13 (green), and undigested CB15, CB2, and CB13 (white). A Lambda BstE digest (black) was used as a size marker with the top band being 8454 bp. Under these electrophoresis conditions, large pieces of cleaved chromosomal DNA migrated to a position that was slightly higher than the 8454 bp band while the uncleaved genomic DNA remained in the wells of the gel.

**Figure 3:**
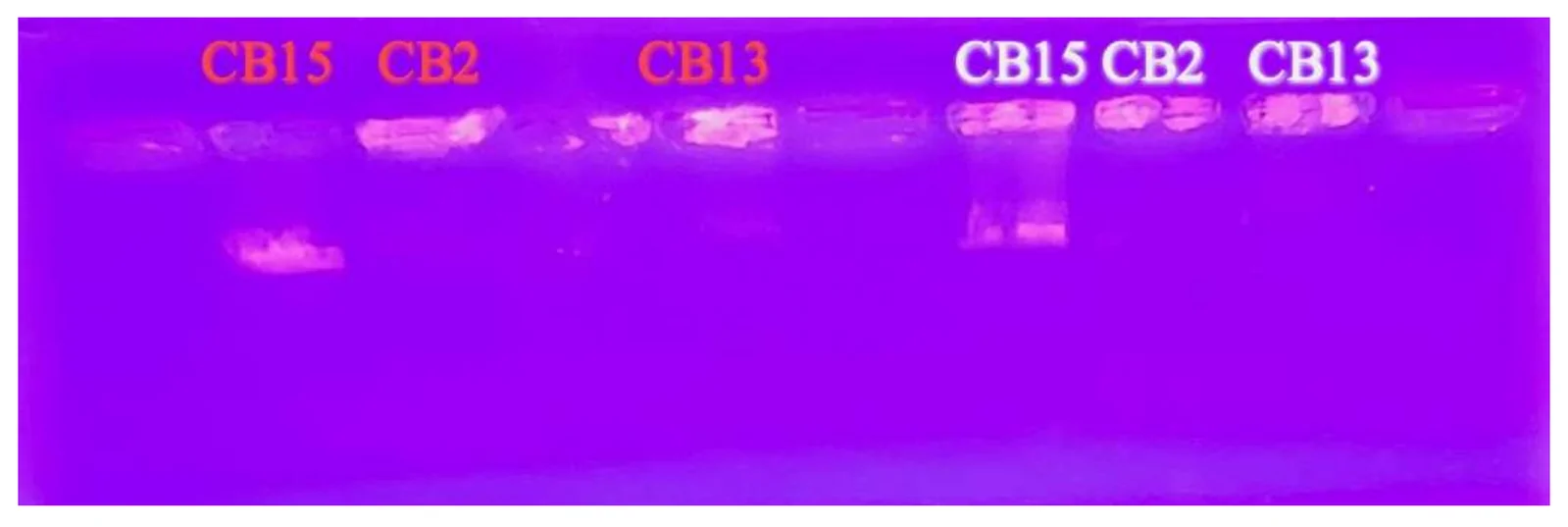
Agarose gel electrophoresis performed on AseI-digested CB15, CB2, and CB13 (red) and undigested CB15, CB2, and CB13 (white).

Since strain CB13 is known to code for a REase that cleaves the DNA at CTCGAG sites, the presence of this restriction enzyme could be responsible for the differences in plating efficiency when CB13 is the host. However, the 5′-CTCGAG-3′ sequence was not present in the ERS, KSC, or Lullwater genomes. Therefore, we hypothesized that the CB2 and CB13 genomes would code for a second RM system that recognized a different restriction site that was present in the ERS and KSC genomes but was not present in the Lullwater genome. To test this hypothesis, we performed an analysis of potential restriction enzyme binding sites and showed that the ERS and KSC genomes contained an NsiI 5′-ATGCAT-3′ restriction site and a ClaI/BspDI 5′-ATCGAT-3′ restriction site, and that neither of these sites was present in the Lullwater genome. To test if the CB2 and CB13 genomes coded for an enzyme that methylated and protected these sites, we performed restriction enzyme digests with NsiI and BspDI on the CB15, CB2, and CB13 genomes. After the digest, the CB2 and CB13 genomes remained uncut, while the genome CB15 was cut (Figure 2). Thus, both CB2 and CB13 synthesized a methylase that protected both restriction sites in contrast to CB15 which did not. Since the two restriction sites differ at only the two central bases, we hypothesized that the RM system shared by the CB2 and CB13 genomes targeted 5′-ATNNAT-3′ sites. To test this hypothesis, we performed a restriction digest with AseI which cuts at 5′-ATTAAT-3′ sites. Figure 3 shows that AseI also failed to cut the CB2 and CB13, providing further support for the hypothesis that ATNNAT was the target site for the newly identified CB2 and CB13 RM systems. However, when we checked the genome annotations for these strains, no other RM systems were identified, suggesting that the new RM system might be a novel restriction system.

To determine if closely related wild-type *Caulobacter* strains also used this CB2/CB13 R/M system, we digested the genomic DNA of *C. vibrioides* strains NA1000, CB1, ME4, FWC26, and *C. segnis* strains CBR1, and TK0059 with the AseI restriction enzyme and found that the NA1000 genomic DNA was cut, but the genomic DNAs from the other strains were not cut. Since the AseI sites are protected in both the *C. vibrioides* and *C. segnis* genomes, it is likely that the same R/M system is present in all of these genomes. In contrast, the AseI sites in the NA1000 genome were not protected so that genome does not code for the ATNNAT RM system. However, we showed that the plating efficiency of KSC phage was reduced 1000-fold when strains NA1000 or CB1 were used as the host. Since the NA1000 strain lacks the ATNNAT RM system and is considered a variant of CB15 [14], the reduced plating efficiency was a surprising result. Marks et al. [14] examined the sequence differences between the CB15 and the NA1000 genomes and identified 10 SNPs and a 26 kb mobile element that was present only in the NA1000 genome. When we tested the strains containing single swaps of each of the 10 SNPs, none of them changed the plating efficiency of either strain. However, when a CB15 strain with the mobile element inserted in its genome was tested, it exhibited the reduced plating efficiency observed with the NA1000 strain. Therefore, one or more of the mobile element genes confers the reduced plating efficiency. The mobile element codes for 23 genes including a conjugation/integrase complex, a pair of HipBA toxin/antitoxin genes, and 11 sugar metabolism genes [14]. Since HipA inactivates glutamyl tRNA synthetase [15,16], we hypothesize that phage infection triggers increased HipA expression which would inhibit phage protein synthesis.

## 4. Discussion

*C. vibrioides* strains CB2 and CB13 appeared to have functional RM systems since growth on either CB2 or CB13 eliminated the lower plating efficiency observed when they were grown on CB15 (Table 1). In contrast, when ERS or KSC was grown on CB2 or CB13, the plating efficiency improved when the phages were plated onto the CB2 and CB13 hosts again. These results suggest that both CB2 and CB13 could methylate the same DNA restriction site to protect the phage DNA in both hosts.

Further analyses confirmed that CB2 and CB13 have a similar RM system. Restriction enzyme digests using three different restriction enzymes that cut 5′-ATNNAT-3′ (a site found many times in ERS and KSC) did not cleave the CB2 or CB13 genomes. Thus, the two strains each code for a novel RM enzyme system that recognizes the 5′-ATNNAT-3′ site. Interestingly, we demonstrated that this restriction site modification is also present in all the additional *C. vibrioides* and *C. segnis* strains that we tested. Therefore, this RM system has been conserved among *Caulobacter* species. However, a functional version of this restriction system is not present in the *C. vibrioides* CB15 or NA1000 genomes, suggesting that it has been lost or inactivated in these two strains.

Although many bacterial strains have well-characterized RM systems, there are no previously known restriction enzymes that recognize all versions of a 5′-ATNNAT-3′ site. In addition, an analysis of the *Caulobacter* genome annotations failed to reveal any restriction enzymes that might recognize the 5′-ATNNAT-3′ site. Therefore, we may have identified a previously undescribed kind of RM system that is present in these *Caulobacter* strains.

Despite the missing RM system in NA1000, we also observed reduced plating efficiency on this strain, and we were able to show that the reduced plating efficiency was due to the presence of the 26 kb mobile element. The genes for a toxin/antitoxin system present in the mobile element may be responsible for the reduced plating efficiency in this strain. Marks et al. [14] characterized the mobile element in the NA1000 strain as an insertion. However, since it is also present at the same location in the CB1 genome, it is more reasonable to assume that the CB15 strain lost the mobile element. Therefore, the early and continued success of the Ely laboratory phage isolation efforts are in part due to the lack of both the NA1000 TA system and the *C. vibrioides* RM system in the CB15 strain that we chose as a phage host.

## Figures and Tables

**Table 1:** Plating efficiencies of phages ERS, KSC, and Lullwater (LWR) on *Caulobacter* strains CB15, CB2, and CB13 when grown on each of the strains as the primary host. The ratios are the average of three independent determinations.

Host	ERS/CB15	KSC/CB15	LWR/CB15	ERS/CB2	KSC/CB2	LWR/CB2	ERS/CB13	KSC/CB13	LWR/CB13
CB15	1	1	1	1	1	1	1	1	1
CB2	0.16	3 × 10^−4^	0.5	0.5	1	1	1.4	0.7	1.1
CB13	2 × 10^−4^	<10^−6^	0.9	0.6	0.8	0.8	34	1	0.9

## Data Availability

The original contributions presented in this study are included in the article. Further inquiries can be directed to the corresponding author.

## References

[1] Ely B., Lenski J., Mohammadi T., Tubman I. (2023). Structural and genomic diversity of bacteriophages. Bacteriophages: Methods and Protocols.

[2] Egido J., Costa A., Aparicio-Maldonado C., Haas P., Brouns S. (2021). Mechanisms and clinical importance of bacteriophage resistance. FEMS Microbiol. Rev..

[3] Vasu K., Nagaraja V. (2017). Diverse Functions of Restriction-Modification Systems in Addition to Cellular Defense. Molec Cell Biol. Rev..

[4] Poindexter J. (1964). Biological properties and classification of the *Caulobacter* group. Bacteriol. Rev..

[5] Syddall R., Stachow C. (1985). A site-specific endonuclease from *Caulobacter crescentus* CB-13: Restriction endonuclease *CcrI*. Biochim. Biophys..

[6] Nierman W.C., Feldblyum T.V., Laub M.T., Paulsen I.T., Nelson K.E., Eisen J.A., Heidelberg J.F., Alley M.R., Ohta N., Maddock J.R. (2001). Complete genome sequence of *Caulobacter crescentus*. Proc. Natl. Acad. Sci. USA.

[7] Scott D., Ely B. (2016). Conservation of the essential genome among *Caulobacter* and *Brevundimonas* species. Curr. Microbiol..

[8] Johnson R., Wood N., Ely B. (1977). Isolation and characterization of bacteriophages for *Caulobacter crescentus*. J. Gen. Virol..

[9] Ely B., Hils M., Clarke A., Albert M., Holness N., Lenski J., Mohammadi T. (2024). New genera and species of *Caulobacter* and *Brevundimonas* bacteriophages provide insights into phage genome evolution. Viruses.

[10] Wilson K., Ely B. (2019). Analyses of four new *Caulobacter* Phicbkviruses indicate independent lineages. J. Gen. Virol..

[11] Ely B., Croft R.H. (1982). Transposon mutagenesis in *Caulobacter crescentus*. J. Bacteriol..

[12] Ely B., Johnson R.C. (1977). Generalized transduction in *Caulobacter crescentus*. Genet.

[13] Dingwall A., Shapiro L., Ely B. (1990). Analysis of bacterial genome organization and replication using pulsed-field gel electrophoresis. Methods Companion Methods Enzymol..

[14] Marks M.E., Castro-Rojas C.M., Teiling C., Du L., Kapatral V., Walunas T.L., Crosson S. (2010). The genetic basis of laboratory adaptation in *Caulobacter crescentus*. J. Bacteriol..

[15] Germain E., Castro-Roa D., Zenkin N., Gerdes K. (2013). Molecular mechanism of bacterial persistence by HipA. Mol. Cell.

[16] Kaspy I., Rotem E., Weiss N., Ronin I., Balaban N.Q., Glaser G. (2013). HipA-mediated antibiotic persistence via phosphorylation of the glutamyl-tRNA-synthetase. Nat. Commun..

